# SnO_2_ hollow nanotubes: a novel and efficient support matrix for enzyme immobilization

**DOI:** 10.1038/s41598-017-15550-y

**Published:** 2017-11-10

**Authors:** Muhammad Zahid Anwar, Dong Jun Kim, Ashok Kumar, Sanjay K. S. Patel, Sachin Otari, Primata Mardina, Jae-Hoon Jeong, Jung-Hoon Sohn, Jong Hak Kim, Jung Tae Park, Jung-Kul Lee

**Affiliations:** 1 Department of Chemical Engineering, Konkuk University, Seoul, 05029 Republic of Korea; 2 Department of Chemical and Biomolecular Engineering, Yonsei University, Seoul, 03722 Republic of Korea; 3 Cell Factory Research Center, Korea Research Institute of Bioscience & Biotechnology (KRIBB), Daejeon, 34141 Republic of Korea

## Abstract

A major challenge in the industrial use of enzymes is maintaining their stability at elevated temperatures and in harsh organic solvents. In order to address this issue, we investigated the use of nanotubes as a support material for the immobilization and stabilization of enzymes in this work. SnO_2_ hollow nanotubes with a high surface area were synthesized by electrospinning the SnCl_2_ precursor and polyvinylpyrrolidone (dissolved in dimethyl formamide and ethanol). The electrospun product was used for the covalent immobilization of enzymes such as lipase, horseradish peroxidase, and glucose oxidase. The use of SnO_2_ hollow nanotubes as a support was promising for all immobilized enzymes, with lipase having the highest protein loading value of 217 mg/g, immobilization yield of 93%, and immobilization efficiency of 89%. The immobilized enzymes were fully characterized by various analytical methods. The covalently bonded lipase showed a half-life value of 4.5 h at 70 °C and retained ~91% of its original activity even after 10 repetitive cycles of use. Thus, the SnO_2_ hollow nanotubes with their high surface area are promising as a support material for the immobilization of enzymes, leading to improved thermal stability and a higher residual activity of the immobilized enzyme under harsh solvent conditions, as compared to the free enzyme.

## Introduction

Owing to the benignity, high selectivity, and good activity of biocatalysts, there has been a global race to industrialize enzymes for the modern world. Nanoparticle-based enzyme immobilization has attracted significant attention in the past few decades for industrial applications. Several types of nanoparticles with distinct structural and morphological features (e.g., nanotubes, yolk shell, spherical, hollow, composite, and pore shell nanoparticles) have been used for the immobilization of enzymes, prolonging the availability of the biocatalysts and resulting in redundant downstream and purification processes^1–5^. Numerous benefits such as improvements in the activity, stability, and reusability of the enzyme can be achieved by their immobilization onto porous nanostructures. However, in most cases, the use of nanoparticles results in decreased enzyme activity and lesser loading. Thus, immobilization techniques such as adsorption, entrapment, encapsulation, and covalent immobilization display a wide variety of behaviors in terms of the activity, selectivity, specificity, and stability of the enzyme^6–8^.

Glucose oxidase (GOx), horseradish peroxidase (HRP), and lipases have numerous industrial applications, including in food preservation, baking, wine production, development of biosensors^9^, medicines, bioremediation, degradation of dyes^10^, pharmaceuticals, leather making, paper production, foods, cosmetics, and biodiesel production^11–13^. Apart from ester synthesis, lipases also play a pivotal role in acidolysis, alcoholysis, hydrolysis, aminolysis, and interesterification reactions^13^. Although they are efficient biocatalysts, use of pure enzymes is associated with several problems such as their sensitivity, limited reusability, cost, and stability, which limit their industrialization^14–16^. To overcome these problems, enzymes have been immobilized onto a variety of supports, including bio-related nanoarchitectures, metal-organic frameworks and hybrids, polymers, and nanomaterials^17–23^. Additionally, several immobilization strategies have been developed to increase the stability and reusability of enzymes^6,24^. After achieving an efficient enzyme immobilization, researchers have sought to improve the various properties of the enzymes and nanoparticles to develop an ideal biocatalyst^8,25^.

SnO_2_ nanoparticles have been previously synthesized by solvothermal, hydrothermal, gel combustion, spray pyrolysis, physical vapor deposition, and sol-gel methods^26–28^. Compared to the SnO_2_ nanoparticles synthesized by the above mentioned methods, SnO_2_ nanotubes prepared by the simple, short, and economical electrospinning method have a well-defined tubular structure, with the lumen opening to the exterior. Additionally, the internal morphology and hydrophobicity of the support significantly affect the activity, stability, specificity, and selectivity of the immobilized enzyme^29,30^. Moreover, SnO_2_ nanoparticles exhibit different levels of electrical, optical, and magnetic properties depending on their particle size^31,32^. A variety of nanotubes and their composites, including carbon, halloysite, and SnO_2_ nanoparticles have previously been used as supports for the immobilization of enzymes^17,33–36^. Therefore, in the present study, unique SnO_2_ hollow nanotubes were synthesized by electrospinning methods and used for the first time as supports for the immobilization of enzymes, including lipase, HRP, and GOx. Compared with other nanoparticles reported previously^17,20,36–40^, the synthesized SnO_2_ hollow nanotubes were found to be superior supports for immobilizing a variety of enzymes owing to their large surface areas, additional surface porosities, and luminal openings. The improved immobilization yield and reusability of the SnO_2_ nanotube-bound enzymes indicate that these hollow nanotubes are promising supports for enzyme immobilization. In addition, enzymes immobilized on SnO_2_ hollow nanotubes exhibited higher loadings and improved properties compared to enzymes immobilized on carbon nanotubes^35,38,39^. Given the improved biochemical properties and stability of the immobilized enzymes, it is reasonable to expect that the SnO_2_ hollow nanotubes would also be a promising candidate for the immobilization of various important enzymes for industrial applications.

## Results and Discussion

### Synthesis and characterization of the SnO_2_ hollow nanotubes

**Figure 1:**
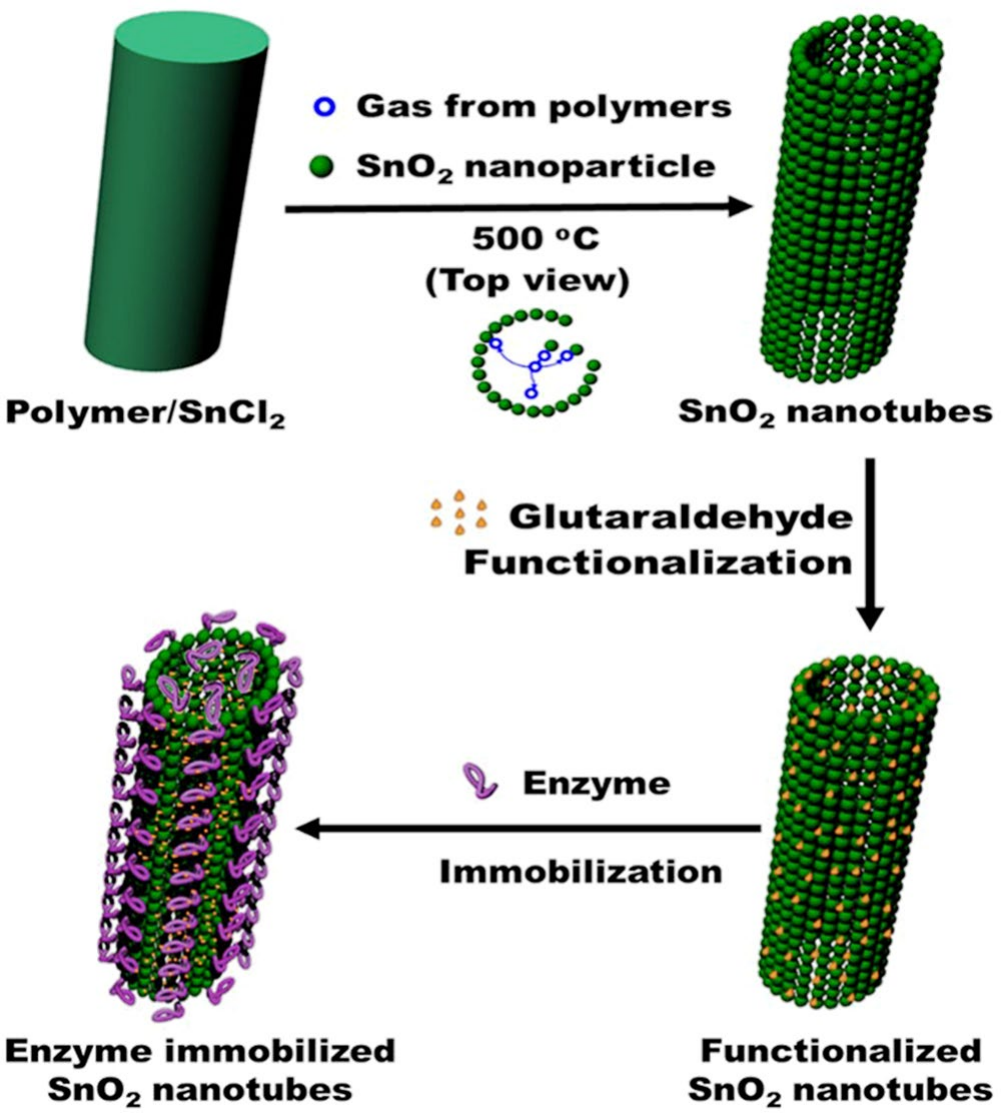
Schematic diagram showing the synthesis of SnO_2_ hollow nanotubes followed by their functionalization and enzyme immobilization.

**Figure 2:**
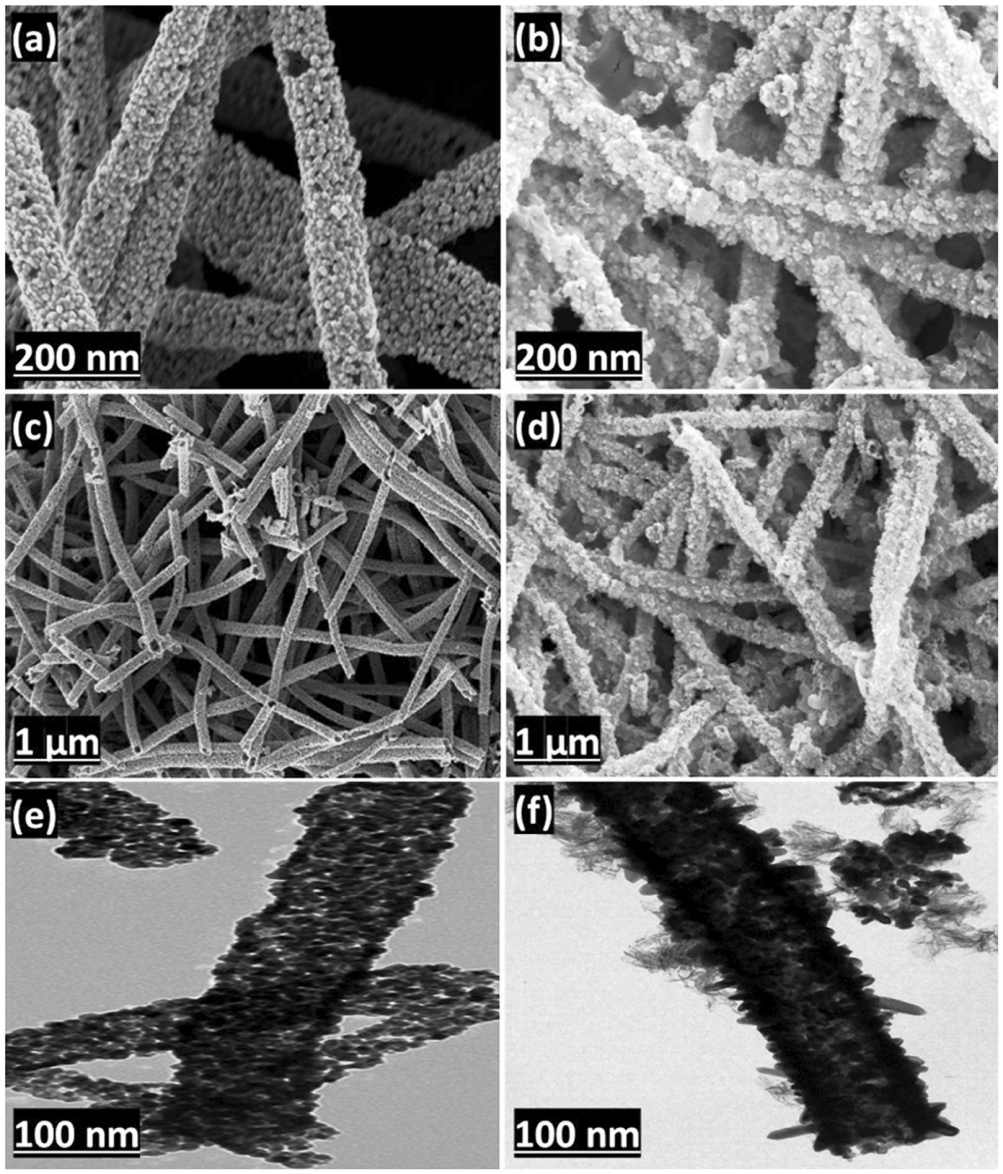
Morphology of the SnO_2_ hollow nanotubes. SEM images (**a**,**c**) before and (**b**,**d**) after enzyme immobilization. TEM images (**e**) before and (**f**) after enzyme immobilization.

**Figure 3:**
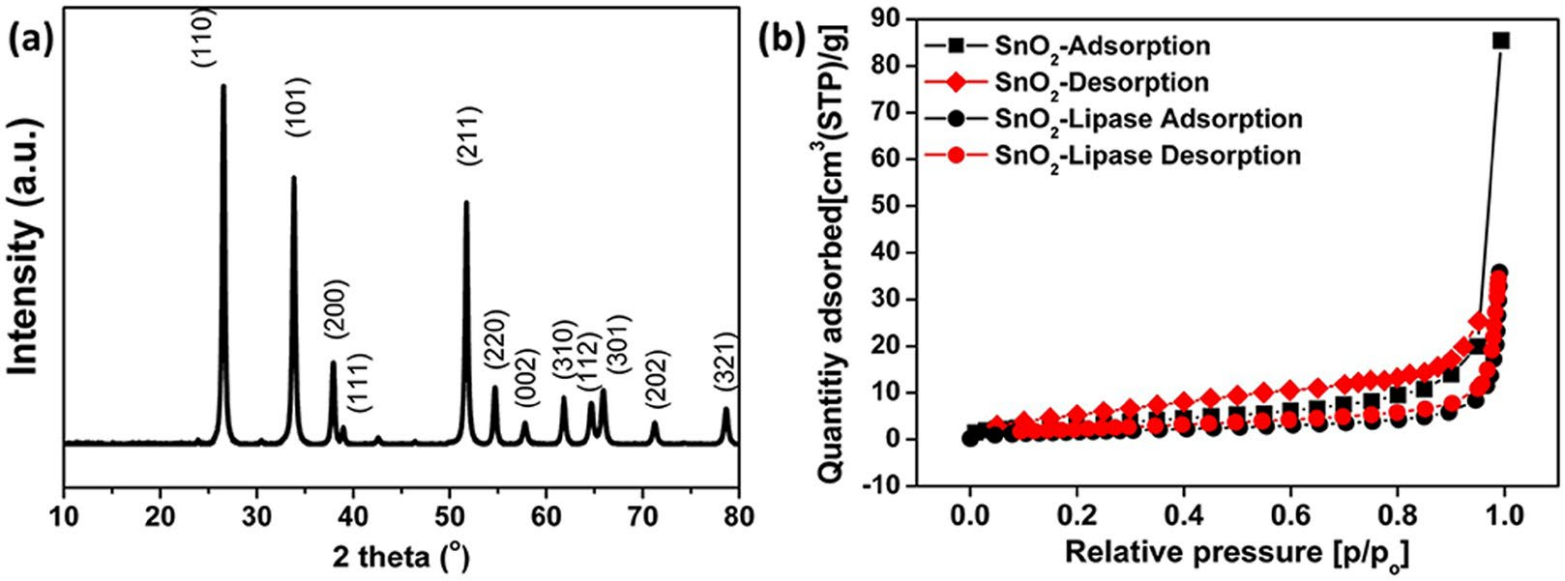
Properties of SnO_2_ hollow nanotubes prepared by the electrospinning method. (**a**) XRD pattern and (**b**) BET isotherm.

A schematic diagram of the synthesis of SnO_2_ hollow nanotubes and the subsequent immobilization of the enzyme is given in Fig. 1. The SnO_2_ hollow nanotubes were prepared by a modified version of a previously reported method^41^. A solution of polyvinylpyrrolidone (PVP, used as the sacrificial agent to form the main pores of the nanotubes) and SnCl_2_ in a mixed solvent solution of dimethyl formamide (DMF) and ethanol was electrospun. The product was dried in an oven and then subjected to thermal annealing in air. This calcination process resulted in the successful fabrication of SnO_2_ hollow nanotubes of length ranging from a few hundred nanometers to a few micrometers and diameter in the range of 200–300 nm. The SnO_2_ hollow nanotubes were composed of clusters of tens of nanometer-sized nanoparticles (Fig. 2). Although the tubular structures could be inferred from the scanning electron microscopy (SEM) images (Fig. 2a and c), transmission electron microscopy (TEM) (Fig. 2e) and the back-scattering mode of SEM were used to confirm the inner vacancy of the structures (Supplementary Fig. 1). The tubular shape instead of a rod-like form indicated that the structure consisted of SnO_2_ nanoparticles, which were ~40–50 nm in size (Fig. 2e). To gain further insight into the crystallographic attributes of the SnO_2_ nanotubes, X-ray diffraction (XRD) was performed (Fig. 3a). All the peaks of the XRD pattern could be assigned to the rutile phase of SnO_2_ (JCPDS No. 41–1445). Furthermore, the size of the SnO_2_ nanoparticles was calculated from the strongest peak (110) using Scherrer’s equation and found to be 43.18 nm, which matched the size evaluated from the SEM images (Fig. 2a and c). The surface area and total pore volume of the SnO_2_ hollow nanotubes were 15.6 m^2^/g and 0.12 cm^3^/g, respectively, as calculated with the multi-point Brunauer–Emmett–Teller (BET) method, and the isotherm was plotted accordingly (Fig. 3b). The surface area and total pore size significantly decreased up to 6.3 m^2^/g and 0.05 cm^3^/g, respectively, upon immobilization of the enzyme on the SnO_2_ hollow nanotubes, indicating both internal and external immobilization. Moreover, as revealed in the Barrett–Joyner–Halenda (BJH) plot (Supplementary Fig. 2), there were small peaks corresponding to the pore sizes of 24.4 and 58.1 nm, indicating the existence of pores among the SnO_2_ nanoparticles. A decrease in the SnO_2_ hollow nanotube porosity to 21.3 and 43.6 nm indicated internal immobilization of the enzyme, which was expected to facilitate the diffusion of the substrate. The presence of larger pores (with diameter over 100 nm) was attributed to the hollow structure of the SnO_2_ nanotubes. Additionally, the average pore diameter deduced from the BJH method for the SnO_2_ nanotubes was 45.4 nm. As globular enzymes like lipase, HRP, and GOx have a typical diameter of 3–6 nm, they could be easily loaded into the lumen of these SnO_2_ nanotubes and it was difficult for them to diffuse back out owing to their covalent binding with the support matrix. The use of SnO_2_ nanotubes as a support resulted in a high loading (217 mg/g) and immobilization yield (93%) of lipase, which could be attributed to the additional porosity along with the luminal openings (evident in Fig. 3b and Supplementary Fig. 2). In comparison with a previous report that indicated an enzyme loading of 11.6 mg/g for pristine halloysite nanotubes (HNTs) and 168.8 mg/g for dopamine surface-modified HNTs, the highly porous SnO_2_ hollow nanotubes synthesized in this work provide a better support for enzyme immobilization^33^.

### Enzyme immobilization

**Figure 4:**
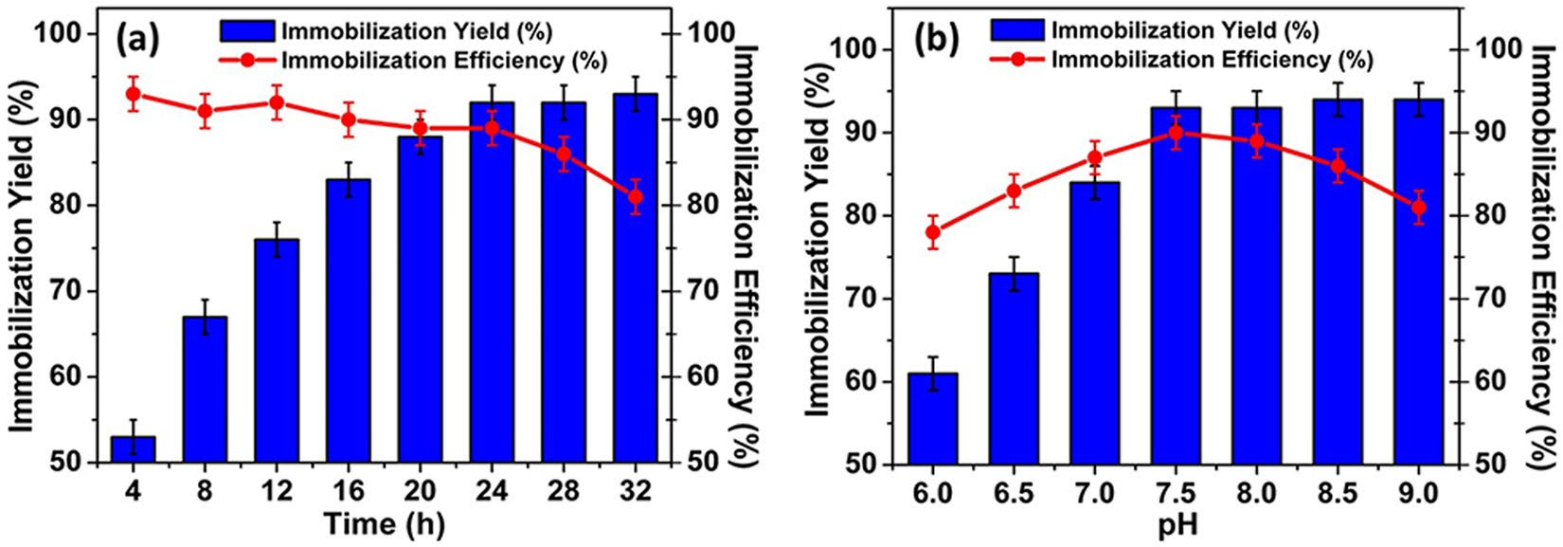
Immobilization of lipase onto SnO_2_ hollow nanotubes. (**a**) Time and (**b**) pH profiles with the immobilization yield (%) and immobilization efficiency (%).

The synthesized particles were functionalized with different concentrations of glutaraldehyde and the immobilization yield (%) was checked versus the immobilization efficiency (%). Glutaraldehyde concentrations of 0.8, 0.6, and 1 M were investigated for optimal functionalization of the SnO_2_ hollow nanotubes for lipase, HRP, and GOx immobilization, respectively. Overall, the immobilization of the enzymes increased over time, and the optimum immobilization efficiency and loading were obtained after 24 h for all three enzymes (Fig. 4a). The pH of the solution had a profound effect on enzyme immobilization. Among the solutions with different pH utilized for optimizing immobilization, the solution with pH 7.5 gave the highest immobilization efficiency and immobilization yield for lipase (Fig. 4b), whereas the optimum pH value for the immobilization of HRP and GOx was 7.0. Moreover, enzyme immobilization at 4 °C resulted in the highest immobilization efficiency and immobilization yield, as depicted in Supplementary Fig. 3 for lipase.

The SnO_2_ hollow nanotubes showed an immobilization yield of 93% and an immobilization efficiency of 89% for lipase. The activities of HRP and GOx were also retained to a substantial extent after immobilization, with immobilization efficiencies of 78% and 81%, respectively (Table 1). The variations in the immobilization parameters may be because of the differences in the properties of the enzymes. It was found that for each 1 g of SnO_2_ hollow nanotubes, the loading of lipase, HRP, and GOx was 217, 181, and 196 mg, respectively.

**Table 1:** Immobilization of enzymes on SnO_2_-nanotubes.

Enzyme	Amount of proteins in wash out	Immobilization Yield (%)	Immobilization Efficiency (%)
Lipase	7.30 ± 2.3	92.7 ± 4.3	88.7 ± 3.4
HRP	18.5 ± 3.1	81.5 ± 6.2	77.6 ± 5.3
GOx	27.4 ± 3.4	72.6 ± 5.6	81.4 ± 6.2

### Leaching and cross-linking of immobilized lipase

Although immobilization can significantly improve the different characteristics of the enzymes, leaching is still a key problem because it hinders the repeated use of the immobilized enzyme^4,42,43^. The SnO_2_ hollow nanotube-immobilized lipase was treated with NaCl to evaluate the leaching effect. A substantial amount of the wash-out protein was detected in the supernatant. This excess amount of the enzyme in the supernatant corresponded to that had physically adsorbed onto the nanotubes, in addition to the amount that had covalently attached to the substrate. In order to solve the leaching problem and to improve the stability and reusability of the enzyme, additional cross-linking was achieved with glutaraldehyde^44,45^. Among the three enzymes, the least amount of leaching (11%) was noticed for lipase after its cross-linking with 0.1 M glutaraldehyde. The other two enzymes also showed a decrease in leaching after cross-linking (Supplementary Table 1). However, a significant decrease in the immobilization efficiency was observed on using higher concentrations of glutaraldehyde (0.2–0.5 M), which may be due to substrate diffusion limitations after the high cross-linking of the enzyme^4^.

### Characterization of the immobilized lipase

**Figure 5:**
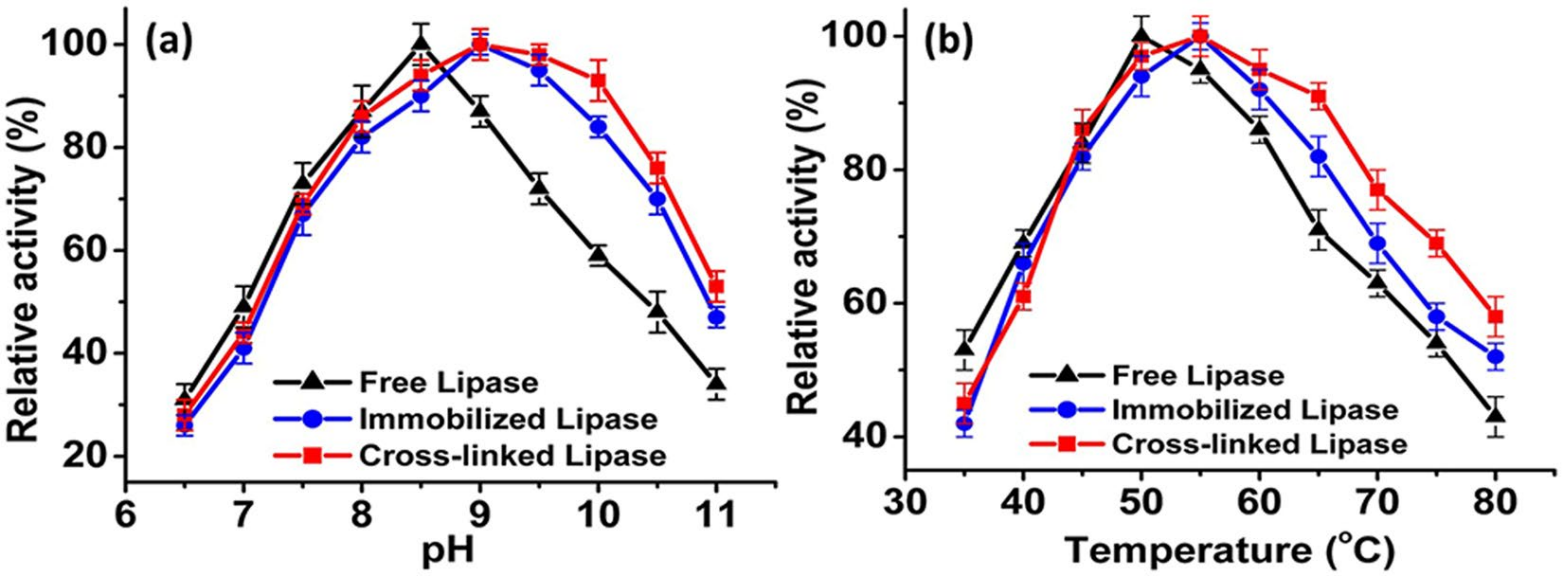
Activity of free lipase and of lipases immobilized and cross-linked onto SnO_2_ hollow nanotubes. At different (**a**) pH and (**b**) temperatures.

Maximum residual activities of the free, immobilized, and cross-linked lipases were obtained at the pH values of 8.5, 9, and 9.5, respectively (Fig. 5a). The immobilized and cross-linked lipases maintained higher residual activities over a broad pH range of 8.5–10 in comparison with the free lipase. The residual activities of the cross-linked lipase were 100%, 98%, and 93% at pH 9, 9.5, and 10, respectively, whereas those of the free enzyme were 87%, 72%, and 59%, respectively. In addition to the shift in the optimum pH values after immobilization, the residual activities of the immobilized and cross-linked lipases were also improved at higher temperatures. The optimum temperature for the free lipase was 50 °C, which shifted to 55 °C for the immobilized and cross-linked lipases (Fig. 5b). At higher temperatures (60–80 °C), the cross-linked lipase retained a higher relative activity in comparison to the free and immobilized lipases. This shift in optimum temperature for the immobilized and cross-linked lipases may be attributed to their binding behavior. The SnO_2_ hollow nanotubes provide additional rigidity to the external backbone of the immobilized enzyme after firmly holding it within their lumen (Fig. 2f). Consequently, the effects usually appeared at higher temperatures (e.g., the breakdown of the interactions responsible for the proper globular and catalytically active structure of the enzyme) became less prominent, leading to enhanced thermal stability of the immobilized enzyme^46^.

### Instrumental analyses of the immobilized enzyme

**Figure 6:**
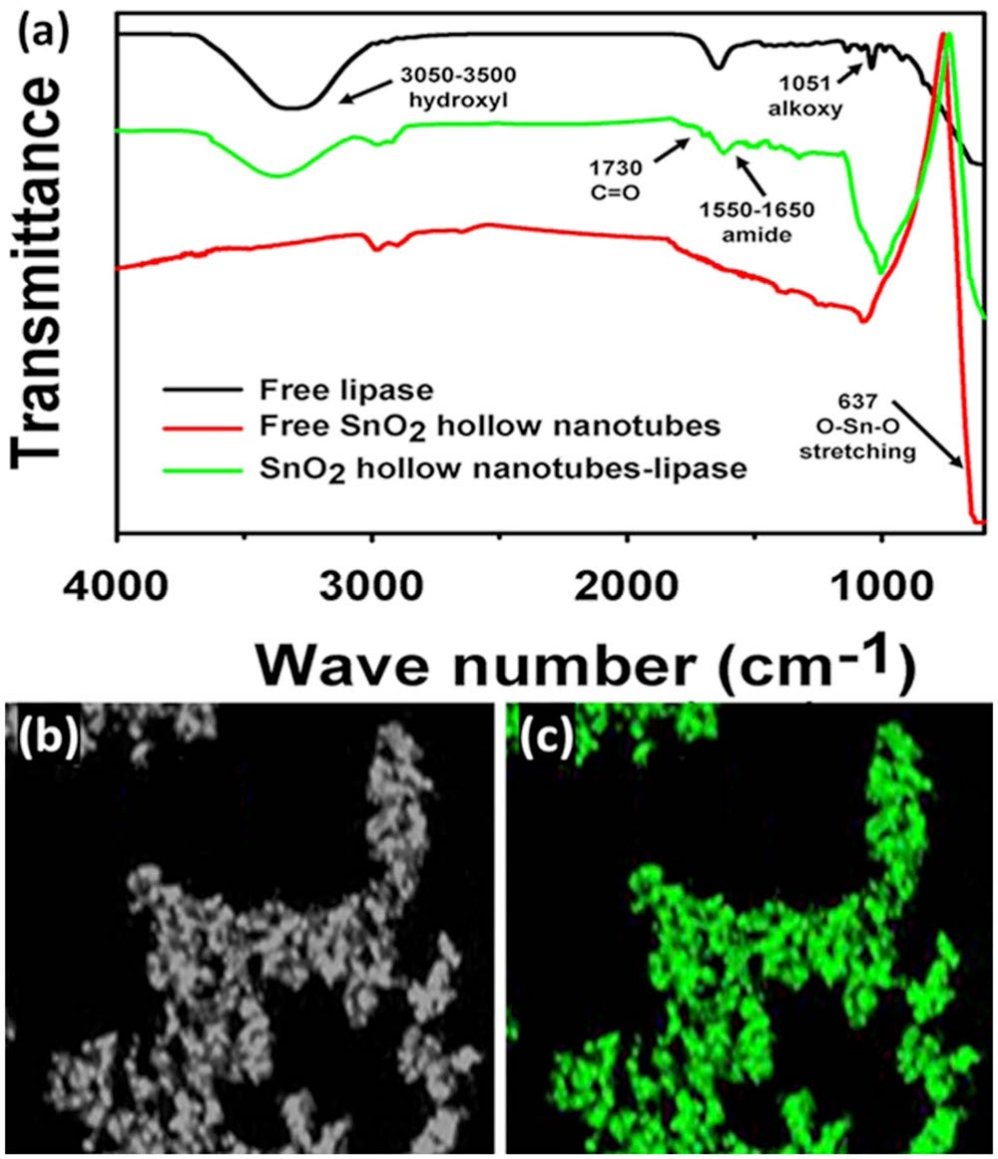
FTIR and confocal laser scanning microscopy (CLSM) results for lipase immobilized on SnO_2_ hollow nanotubes. (**a**) FTIR results for free lipase and for free and lipase-immobilized SnO_2_ nanotubes. (**b**) CLSM image of SnO_2_ hollow nanotubes. **(c)** CLSM image of FITC-stained immobilized lipase.

The SEM images of the immobilized lipases are presented in Fig. 2. It is evident that the immobilized enzyme is bound to the SnO_2_ hollow nanotubes (Fig. 2b and d). The TEM images of the immobilized lipases are provided in Fig. 2e and f. The dark internal background of the SnO_2_ hollow nanotubes after enzyme immobilization confirms the internal localization of lipase. These results suggest that lipase was bound both on the surface and in the internal lumen of the SnO_2_ hollow nanotubes, owing to the porous nature of the support (Fig. 2b,d and f). In order to explore and quantify the SnO_2_ hollow nanotube modifications before and after enzyme immobilization, elemental analysis was performed (Supplementary Table 2). A significant increase in the relative content of each element (C, O, and N) after enzyme immobilization indicated a high loading of the enzyme onto the SnO_2_ hollow nanotubes. Moreover, the high intensity of the green fluorescence on the surface of the SnO_2_ hollow nanotubes was a result of the fluorescein isothiocyanate (FITC)-tagged lipase, indicating a greater concentration of the enzyme on the surface (Fig. 6). A uniform distribution of the enzyme along the walls of the nanotubes was indicated by the green fluorescence (Fig. 6b and c). Thus, on the basis of the SEM, TEM, and confocal laser scanning microscopy (CLSM) results, it was concluded that the enzyme had been immobilized both internally and externally onto the SnO_2_ hollow nanotubes. In addition, the high loading of lipase was confirmed, with a significant reduction in weight to 59% observed for the SnO_2_ hollow nanotubes-lipase complex in the thermogravimetric analysis (Supplementary Fig. 4). The presence of additional peaks at 1718–1613 cm^−1^ in the Fourier-transform infrared (FTIR) spectrum of the immobilized lipase, which could be correlated to the amide bond (N=C=O) stretching vibrations, further illustrated the efficient immobilization of the enzyme (Fig. 6a). The O–Sn–O stretching vibrations of the SnO_2_ nanotube particles were detected at 637 cm^−1^ ^47–49^. The peaks in the range of 1400–757 cm^−1^ in the FTIR spectrum of SnO_2_-bound lipase were attributed to the C–O, C–N, and C–C vibrations. Broad bands detected at 1550–1650 cm^−1^ are consistent with the C=O stretching (amide I band, 1650 cm^−1^) and N-H bending (amide II band, 1550 cm^−1^) vibrations of the protein, with the additional C=O stretching peak (1730 cm^−1^) confirming lipase immobilization^50^. Moreover, the peaks between 3050 and 3500 cm^−1^ could be assigned to the –OH vibrations of amino acids^51^. The secondary structure of lipase immobilized on SnO_2_ hollow nanotubes was examined by circular dichroism (CD). The immobilized lipase exhibited a similar CD spectrum to its free form, suggesting that no significant change to its secondary structure occurs upon immobilization (Supplementary Fig. 5).

### Enzyme kinetics

The kinetic parameters (*K*_m_ and *V*_max_) of free lipase changed after its immobilization onto the SnO_2_ hollow nanotubes (Table 2). The Michaelis–Menten model was applied for the determination of these parameters. The apparent *K*_m_ and *V*_max_ values for free lipase were 0.71 mM and 217 µmol·min^−1^·mL^−1^, respectively. After immobilization, a slight increase of 6.6% and 12.4% in the *K*_m_ value, and a decrease of 11.2% and 16.6% in the *V*_max_ value, were noticed for the immobilized and cross-linked lipases, respectively. This increase in *K*_m_ and decrease in *V*_max_ after immobilization onto the SnO_2_ hollow nanotubes is indicative of substrate inhibition or diffusional hindrances. After enzyme immobilization, most of the nanoparticle surface, lumen, and pores are occupied by the enzyme, which provides a low surface area for substrate interaction. The opposite phenomenon (i.e., a decrease in *K*_m_ and an increase in *V*_max_) appeared when the enzyme loading was decreased to half of the original loading (Supplementary Table 3). This further supports the rationale that the porosity and nanoparticle surface area would decrease with heavy enzyme loading.

**Table 2:** Kinetic parameters for the free, immobilized, and cross-linked lipase.

Lipase	K_m_ (mM)	V_max_ (µmol min^−1^ mL^−1^)
Free	0.71 ± 0.05	217 ± 20
Immobilized	0.76 ± 0.04	195 ± 17
Cross-linked	0.81 ± 0.06	186 ± 10

### Effect of metal ions and organic solvents

The effect of the presence of different metal ions in 2 mM concentration on the activity of the free and immobilized lipases was investigated. Divalent ions, especially Ca^2+^ and Mn^2+^, increased the activity of the immobilized lipase by 62% and 45%, respectively. Although the use of most of the other metal ions led to similar residual activities, addition of Na^+^ and K^+^ ions resulted in a sharp decrease in the activity of the free enzyme (Supplementary Table 4). Using solvents such as dimethyl sulfoxide (DMSO), acetone, *n*-hexane, and DMF resulted in a significant increase of 168%, 152%, 144%, and 117%, respectively, in the immobilized lipase activity. In contrast, phenol, methyl formate, acetic acid, and sodium dodecyl sulfate reduced the activity of the enzyme. An increase in the relative activity (%) was recorded for nonpolar hydrophobic solvents, and vice versa for polar hydrophilic solvents (Supplementary Table 5).

### Thermal, pH stabilities and reusability of the enzyme

**Figure 7:**
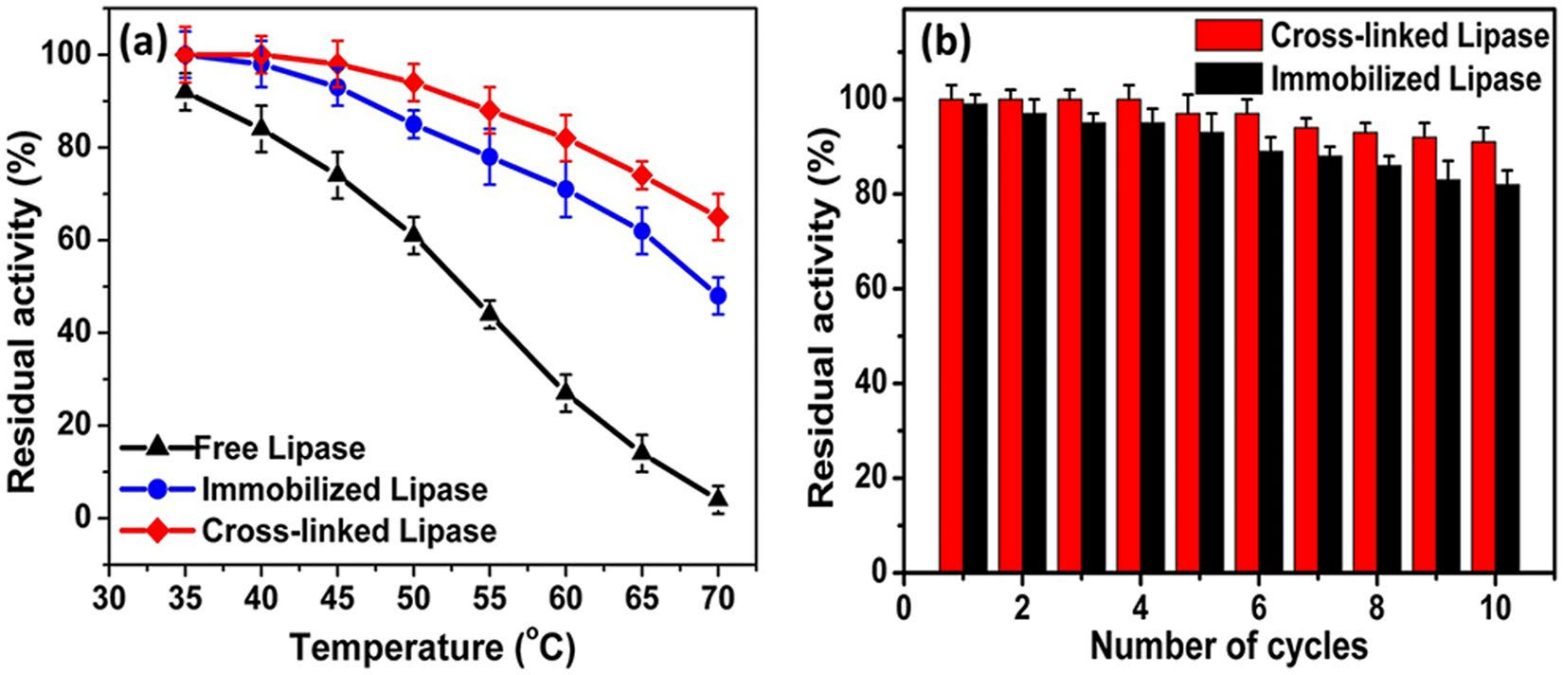
Stability and reusability of free lipase and of lipases immobilized and cross-linked onto SnO_2_ hollow nanotubes. (**a**) Stability at different temperatures (from 35 to 70 °C) and (**b**) reusability.

The thermal stability of an enzyme is of utmost importance for an evaluation of its industrial applicability. Weak forces of attraction between the enzyme and the support particle typically result in poor operational stability, and therefore, covalent immobilization has been introduced to achieve a more firm binding and better stability^34,52^. The stabilities of the free, immobilized, and cross-linked lipases at different temperatures (35–70 °C) were recorded after 4.5 h (Fig. 7a). It was found that the stability of the immobilized and cross-linked lipases at 70 °C increased from 4% (free lipase) to 42% and 61%, respectively, after incubation for 4.5 h, which was almost 11- and 15-fold higher than that of free lipase (Supplementary Fig. 6). The half-lives of the free, immobilized, and cross-linked lipases at 50 °C were 7.1, 22, and 31 h, respectively (Supplementary Fig. 6). The half-life of the cross-linked lipase was 141% and 333% higher than that of the immobilized and free lipases, respectively. Overall, the thermal stability of the cross-linked lipase was significantly enhanced after cross-linking with glutaraldehyde. Compared with the results of a previous report, in which a Fe_3_O_4_@chitosan-bound lipase retained 53% of its initial activity after 3 h incubation at 70 °C, the SnO_2_ hollow nanotube-immobilized lipase showed 150% higher relative stability at the same temperature^53^. In comparison with yet another study where a hen egg derivative-immobilized lipase showed a half-life of 2 h at 70 °C, the immobilized lipase in our study presented 225% higher relative stability at the same temperature^4,45^. The enhanced thermostability of the immobilized and cross-linked lipases described in this work may be accredited to their binding mode with the SnO_2_ hollow nanotubes, in which the carrier provided an external backbone to the enzyme to avoid its structural deformation and thus the negative effects of temperature were minimal (Figs 2f and 3b). In addition to its thermal stability, the immobilized lipase showed a significant increase in its pH stability. The immobilized and cross-linked lipases showed 38% and 52% stability at pH 11 after 4 h, whereas the free enzyme completely lost its residual activity (Supplementary Fig. 7). At an alkaline pH, the immobilized and cross-linked lipases were more stable compared to the free lipase. This increase in pH stability may be attributed to the binding behavior of the lipase with the SnO_2_ hollow nanotubes that maintains the three-dimensional structure of the enzyme even at elevated pH values and resists the interference of high pH^54^.

The reusability of an enzyme also plays a pivotal role in its industrial application. As indicated in Fig. 7b, the cross-linked and immobilized lipases retained 91% and 82% of their original activities after ten consecutive cycles of use, where the cross-linked lipase had better performance in this regard. The loss of activity of the cross-linked and immobilized lipases can be attributed to the weakening of the binding between the enzyme and the support. Moreover, frequent encounters between the active site and the substrate may distort the immobilized enzyme, resulting in its decreased catalytic efficiency. In previous studies, the reported loss in residual activity of immobilized lipase was 12% after the first cycle^35^, 55% after seven cycles^45^, and 30% after ten repetitive cycles^53^.

### Electrochemical analysis

The cyclic voltammograms of the SnO_2_ hollow nanotubes and the free and immobilized lipases affixed onto a glassy carbon electrode (GCE) in a buffer (50 mM) containing palm oil (50 mg/dL), are presented in Supplementary Fig. 8. The anodic and cathodic current peaks for palm oil were detected in the presence of a tri-electrode biosensor. The bioelectrode showed reduction potentials of 0.49, 0.58, and 0.71 V for the free SnO_2_ hollow nanotubes, free lipase, and SnO_2_ hollow nanotube-immobilized lipase, respectively. Here, the shift in the redox potential of palm oil for the immobilized lipase might be primarily due to the charge-conducting SnO_2_ hollow nanotubes. The maximum peak current densities for the free and immobilized lipases in the presence of 50 mg/dL of palm oil were 0.22 and 1.54 µA cm^−2^, respectively. With these highly desirable electrochemical properties, the results suggest that the immobilized lipase is quite efficient at detecting long-chain fatty acids like palm oil. The high recognition ability of the lipase toward long-chain fatty acids after its immobilization onto SnO_2_ nanotubes may be attributed to the conformational changes in the enzyme structure that lead to lid domain opening, which facilitates enzyme–substrate interactions. The optimum pH values for the maximum anodic currents of the free and immobilized lipases fixed onto the GCE were pH 8.0 and 8.5, respectively. The current density increased linearly with an increase in the substrate concentration from 25 to 100 mg/dL. Here, the immobilized lipase was more efficient for the detection of palm oil compared to the free enzyme. Thus, this work presents the first example of an enzyme immobilized onto SnO_2_ hollow nanotubes and its use as a biosensor.

## Conclusions

In this study, SnO_2_ hollow nanotubes with a high surface area were prepared by the electrospinning method. The unique properties of the SnO_2_ hollow nanotubes were employed as a support for the immobilization of lipase, GOx, and HRP. Based on the higher loading capacities and residual activities of the enzymes after their immobilization, it is concluded that SnO_2_ hollow nanotubes are an efficient support for enzyme immobilization. Moreover, cross-linking significantly enhanced the properties of the enzymes, such as their stability at elevated temperatures. Additionally, the high stability of the SnO_2_ hollow nanotube-immobilized enzymes, both in commonly used solvents and at high pH values, makes them a suitable alternative biocatalyst for a broad range of applications. Therefore, the immobilization of important enzymes on SnO_2_ hollow nanotubes (with enhanced electrochemical properties) as a support is a promising approach for numerous industrial applications.

## Experimental Section

### Materials and reagents

Lipase from *Thermomyces lanuginosus* (Lipozyme TL 100 L) was procured from Novozymes Inc. (Franklinton, NC, USA). HRP and GOx were obtained from Sigma-Aldrich (St. Louis, MO, USA). *p*-Nitrophenyl palmitate (*p*-NPP), pyrogallol, glucose, *o*-dianisidine, hydrogen peroxide, Triton X-100, DMSO, DMF, acetone, and acetic acid were also purchased from Sigma-Aldrich. All other materials were of analytical grade and obtained from commercial sources, unless otherwise stated.

### Synthesis of SnO_2_ hollow nanotubes

The SnO_2_ hollow nanotubes were prepared by following a modified version of a previously reported method^41^. A specific amount of SnCl_2_ (0.4 g) was dissolved in a 1:1 solvent mixture of DMF (2.5 mL) and ethanol (2.5 mL). After the solution had become homogeneous, 0.5 g of PVP (*M*_W_ = ~130 M) was dissolved in the above solution at 50 °C for 3 h. The solution was then loaded into a syringe and electrospun at 0.5 mL/h at 14–15 kV. The collected electrospun product was dried overnight in an oven at 120 °C to evaporate any residual solvent. Finally, the product was thermally annealed in air at 500 °C for 1 h to remove the PVP and to form SnO_2_. The as-prepared SnO_2_ hollow nanotubes were characterized by SEM, TEM, XRD, and BET analyses.

### Enzyme assay

The lipase activity was measured using *p*-NPP as the substrate, according to a previously described method^42^. The assay was performed at 50 °C in a total reaction volume of 200 µL that contained 176 µL of the Tris-HCl buffer (20 mM, pH 8.5) and 20 µL of *p*-NPP. The activity of HRP was determined by using hydrogen peroxide (0.97 M) and pyrogallol (13 mM) as substrates in sodium phosphate buffer (0.1 M) at pH 6. The GOx assay was performed with a HRP (20 µg/mL)-coupled reaction, using *o*-dianisidine (0.32 mM) and glucose (0.1 M) as the substrates in phosphate buffer (0.24 M) at pH 5.6^43^.

### Enzyme immobilization

For the immobilization of enzymes onto the SnO_2_ hollow nanotubes, 10 mg of the nanoparticles were functionalized with 0.6 M glutaraldehyde. The nanoparticles were then mixed with 1 mg of the enzyme (GOx, HRP, or lipase) at different pH and incubated at 4 °C and 150 rpm for 24 h. The unbound enzyme molecules were separated into the supernatant by centrifugation at 13000 rpm for 10 min at 4 °C. The concentration of proteins in the supernatant was measured by the Bradford assay with bovine serum albumin as the standard^55^. The immobilization yield and immobilization efficiency were calculated as described by Patel *et al*.^56^. The synthesized SnO_2_ hollow nanotubes not only provided a profound surface area for the immobilization of the enzyme but also increased the stability and activity of the enzyme under various harsh conditions.

### Optimization of the incubation time

To determine the optimal duration for an efficient immobilization of enzymes onto the SnO_2_ hollow nanotubes, the amount of enzyme immobilized on the particles was determined at specific time intervals. Different concentrations of the enzyme (10–400 mg of enzyme per gram of support) were used to determine the loading capacity of the SnO_2_ hollow nanotubes in the reaction. As lipase demonstrated the highest immobilization efficiency and immobilization yield, it was selected for further study.

### Leaching and cross-linking of enzymes

The leaching of immobilized enzymes on pre-functionalized SnO_2_ hollow nanotubes was determined under similar conditions by treatment with NaCl (1 M) for 1 h at 25 °C. The detached enzymes were monitored by analyzing the protein concentration in the supernatant after centrifugation at 10000 rpm for 10 min at 4 °C. The extent of leaching was determined as described by Patel *et al*.^56^. The immobilized enzymes were cross-linked, using glutaraldehyde (0.1 M) in phosphate buffer (50 mM, pH 7), for 30 min at 4 °C with shaking at 150 rpm.

### Instrumental analyses

The surface structures of the SnO_2_ hollow nanotubes were characterized by SEM (SUPRA 55VP; Carl Zeiss, Oberkochen, Germany). For the TEM measurements, the dried films were first immersed in ethanol and a drop of this solution was placed onto a standard copper grid. The solvent was then allowed to evaporate in air. The XRD experiment was performed on a Rigaku 18 kW rotating anode X-ray generator operated at 40 kV and 300 mA. The measurements were performed using Cu Kα radiation (λ = 1.5406 Å) in the 2θ range of 10°–80° with a scanning speed of 3°/min, and the distance between the sample and the detector was 185 mm. The specific surface area of the SnO_2_ hollow nanotubes was determined from the N_2_ adsorption–desorption isotherm by the BET method (for specific surface area) using a Belsorp-mini II analyzer after drying the sample at room temperature for a day in a vacuum oven. Prior to the measurements, the SnO_2_ hollow nanotubes were degassed at 70 °C under dynamic vacuum (10^−2^ Torr) for 1 h. The total carbon, nitrogen, and oxygen contents of the SnO_2_ hollow nanotubes before and after lipase immobilization were analyzed using the 2400 Series II CHNS/O Elemental Analyzer (100 V; PerkinElmer, Waltham, MA, USA). Both free and immobilized enzymes were characterized by FTIR spectroscopy. In the CLSM analysis, DMSO (2 mg/mL) was used for the dissolution of 8 mg of FITC to label 1 mL of lipase in carbonate buffer (0.5 M, pH 9.0) and the resulting solution was incubated at 300 rpm for 6 h in the dark. The excess FITC was removed by dialysis against distilled water. The FITC-labeled lipase was then immobilized onto the SnO_2_ hollow nanotubes. CLSM images were taken with an FV-1000 Olympus confocal microscope. The organic matter attached to the SnO_2_ hollow nanotubes was detected by thermogravimetric analysis. CD analysis was performed using a CD detector (Chirascan-plus, Applied Photophysics, UK).

### Characterization of the immobilized enzyme

The effect of pH on the activity of the free, immobilized, and cross-linked lipases was determined by using Tris-HCl buffer at different pH. Moreover, the effect of temperature on the activity of each type of lipase was determined at 35–80 °C. Similarly, the effects of different metal ions, organic solvents, and surfactants on both free and immobilized enzyme activities were determined.

### Enzyme kinetics

For determination of the kinetic parameters, the sample was incubated at the optimum temperature and pH with different concentrations of the substrate (0.05–5.0 mM). The kinetic parameters *K*_m_ and *V*_max_ were calculated by nonlinear regression fitting of the Michaelis–Menten equation using Prism 5 (GraphPad Software Inc., La Jolla, CA, USA).

### Stability of free and immobilized enzymes

To investigate the thermal stability of the free, immobilized, and cross-linked lipases, the residual activity (%) of each enzyme was calculated after 4.5 h of incubation at different temperatures (35–70 °C) at pH 7.0. For this purpose, the samples were taken at every 30-min interval and the residual activity (%) was measured under standard assay conditions. The initial activity was taken as 100% in each case. Additionally, the stability of the free, immobilized, and cross-linked lipases was investigated by calculating the residual activity in each case after incubation for 4 h at different pH at the optimal temperature.

### Reusability of the immobilized enzyme

The reusability of the enzyme was determined for both immobilized and cross-linked lipases under standard conditions. After each cycle, the enzyme-bonded nanoparticles were separated by centrifugation at 13000 rpm for 5 min and 4 °C, washed twice with the buffer, and then resuspended in the fresh buffer/substrate solution under standard assay conditions. The residual activity (%) in the first cycle was taken as 100%.

## Electronic supplementary material

Supplementary information
