# Supplementary material for: SnO2 hollow nanotubes: a novel and efficient support matrix for enzyme immobilization
Source: Sci Rep. 2017 Nov 10;7:15333. doi: 10.1038/s41598-017-15550-y (PMC5681633; doi:10.1038/s41598-017-15550-y)
Supplement: Supplementary file 1 — Supplementary information [file 41598_2017_15550_MOESM1_ESM.doc]

Supplementary information

**SnO_2_ hollow nanotubes: a novel and efficient support matrix for enzyme immobilization**

**Muhammad Zahid Anwar^1‡^, Dong Jun Kim^2‡^, Ashok Kumar^1^, Sanjay K.S. Patel^1^, Sachin Otari^1^, Primata Mardina^1^, Jae-Hoon Jeong^1^, Jung-Hoon Sohn^3^, Jong Hak Kim^2^, Jung Tae Park^1^*, Jung-Kul Lee^1^***

#

^1^Department of Chemical Engineering, Konkuk University, Seoul 05029, Republic of Korea

^2^Department of Chemical and Biomolecular Engineering, Yonsei University, Seoul 03722, Republic of Korea

^3^Cell Factory Research Center, Korea Research Institute of Bioscience & Biotechnology (KRIBB), Daejeon 34141, Republic of Korea

**^‡^**These authors equally contributed to this study

^*^Author for correspondence: jkrhee@konkuk.ac.kr

^*^Author for correspondence: jtpark25@konkuk.ac.kr


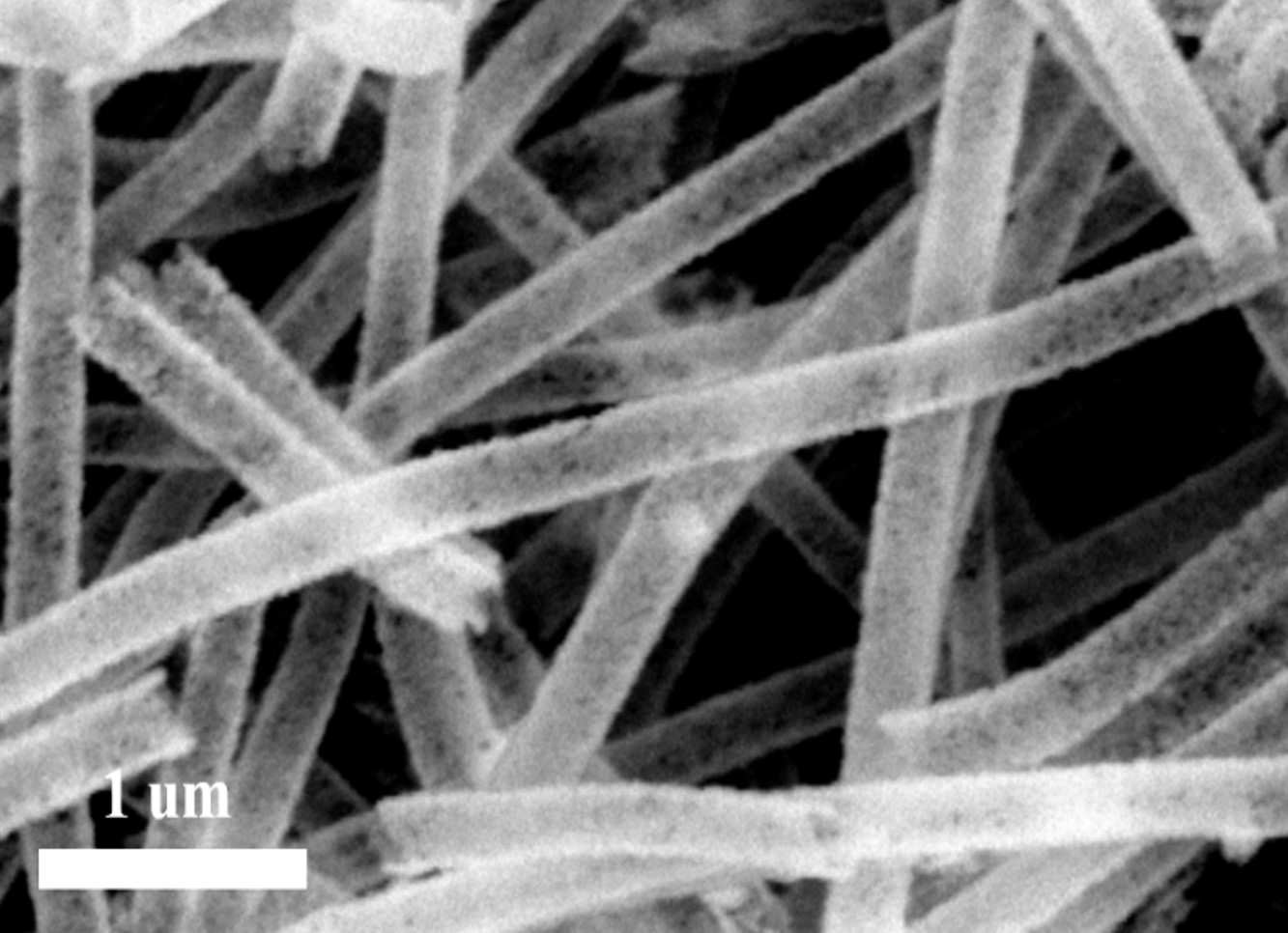


**Supplementary Figure 1.** Back scattering SEM for SnO_2_-nanotubes.


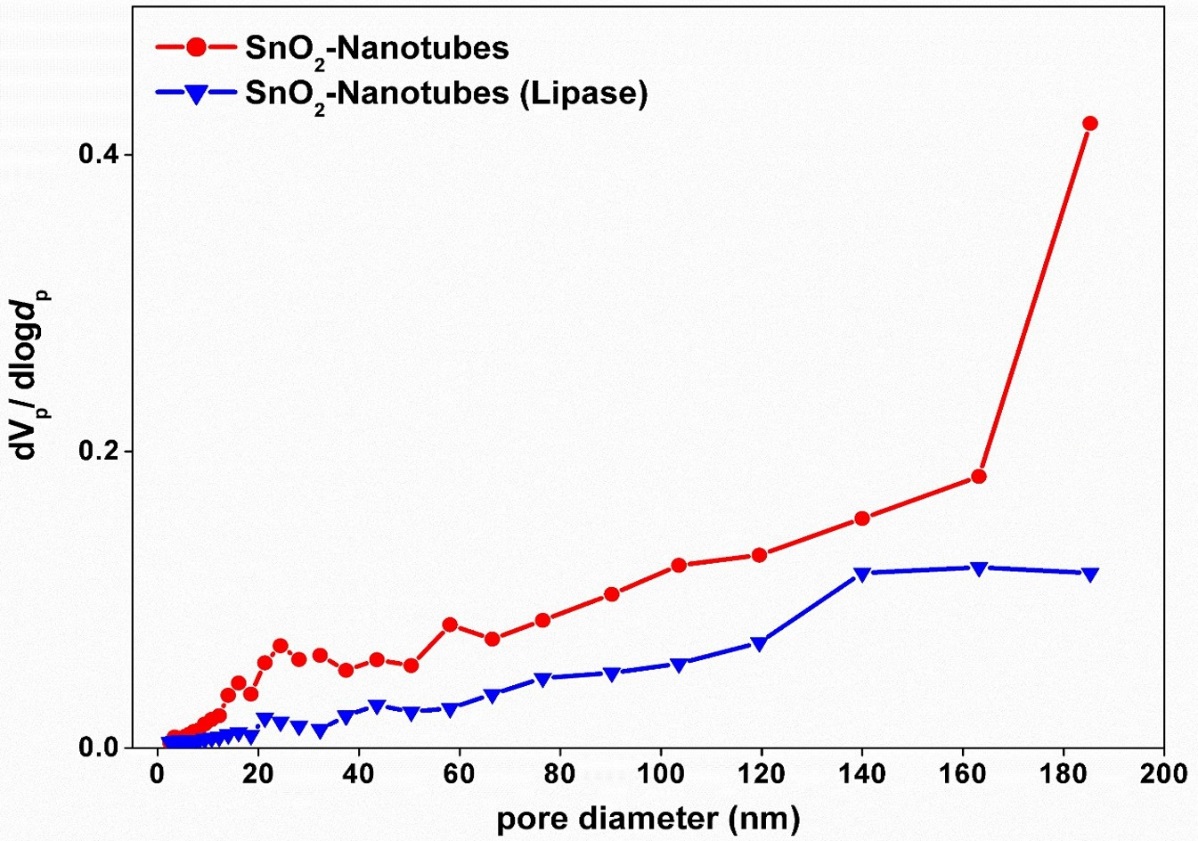


**Supplementary Figure 2.** BJH plot, measuring porosity of SnO_2_ nanoparticles and calculating pore size in hollow structure of SnO_2_ nanotube.


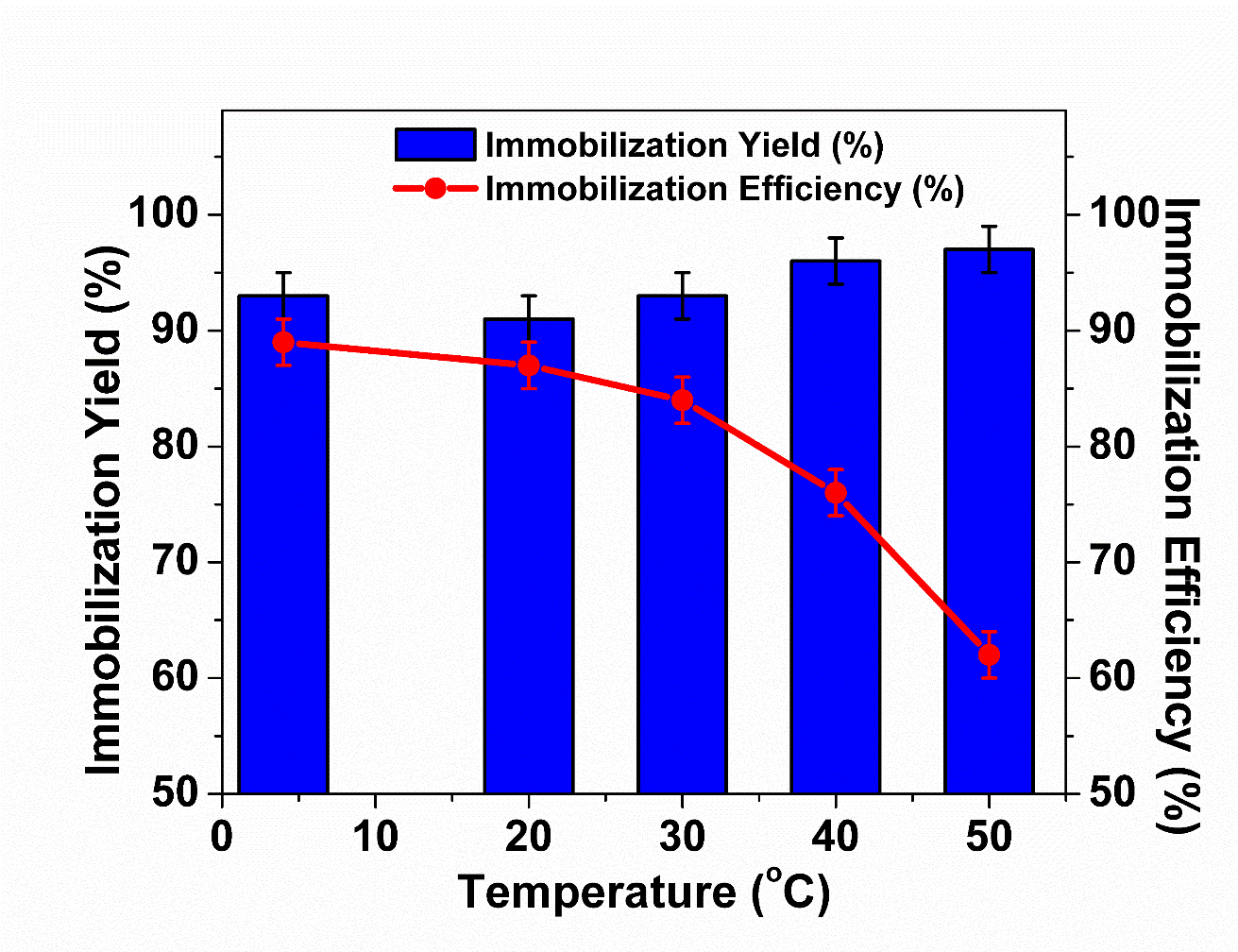


**Supplementary Figure 3.** Immobilization of lipase on SnO_2_-nanotubes, Temperature optimization.


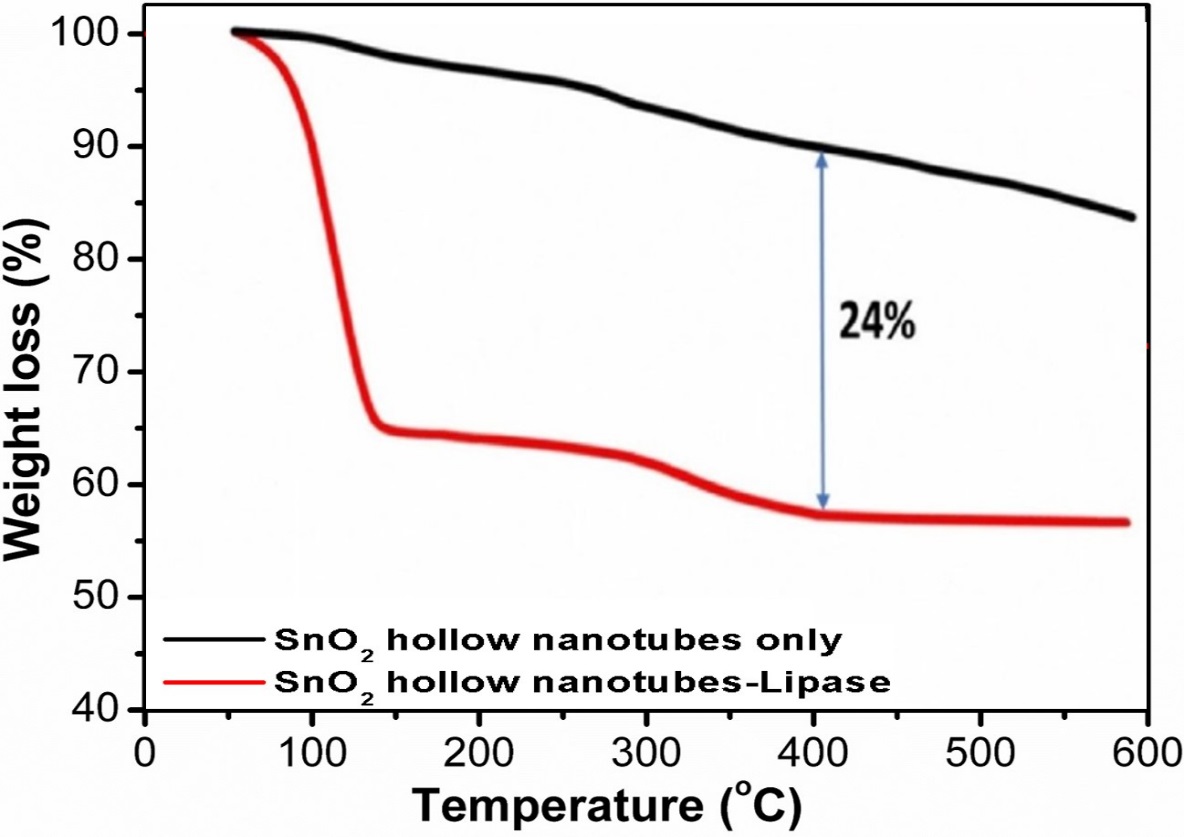


**Supplementary Figure 4.** Thermogravimetric analysis for weight loss in SnO_2_ nanotubes after enzyme immobilization.

**
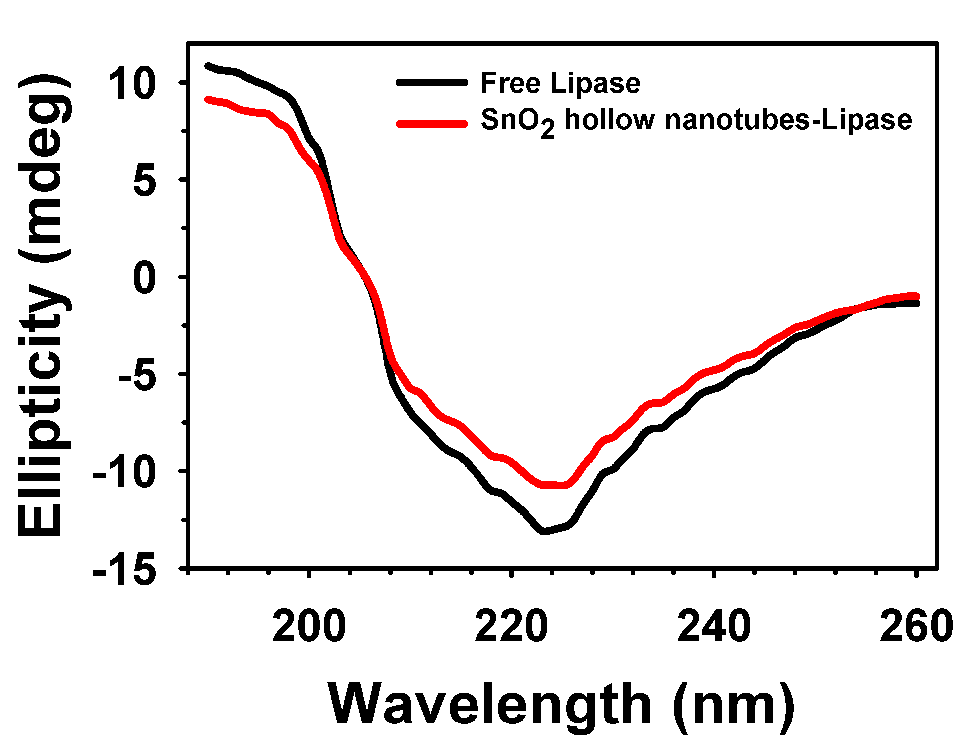
**

**Supplementary Figure 5.** CD analysis of free and immobilized lipase on SnO_2_ hollow nanotubes.

**
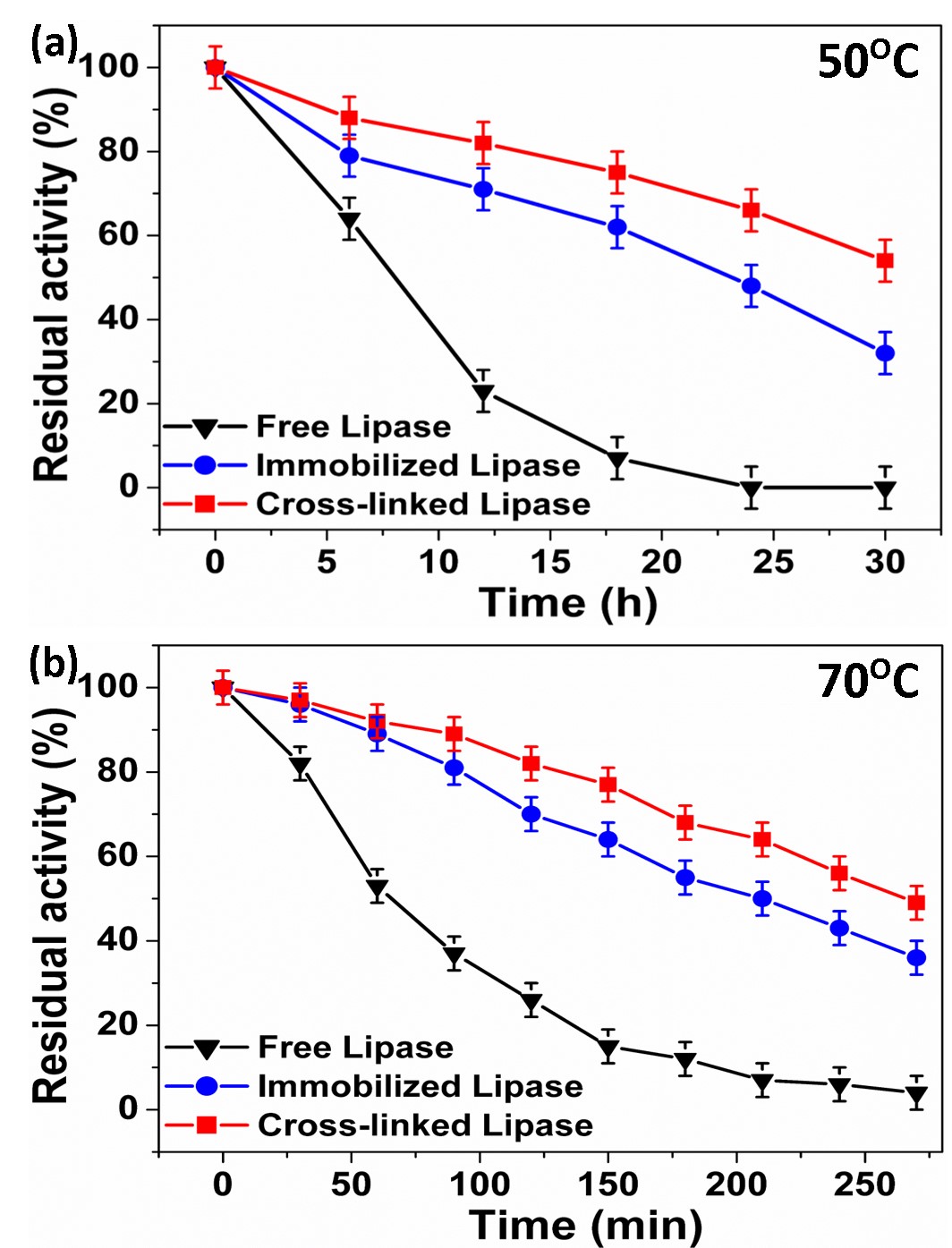
**

**Supplementary Figure 6.** Stability at 50°C and 70°C for free, immobilized and cross-linked lipase.


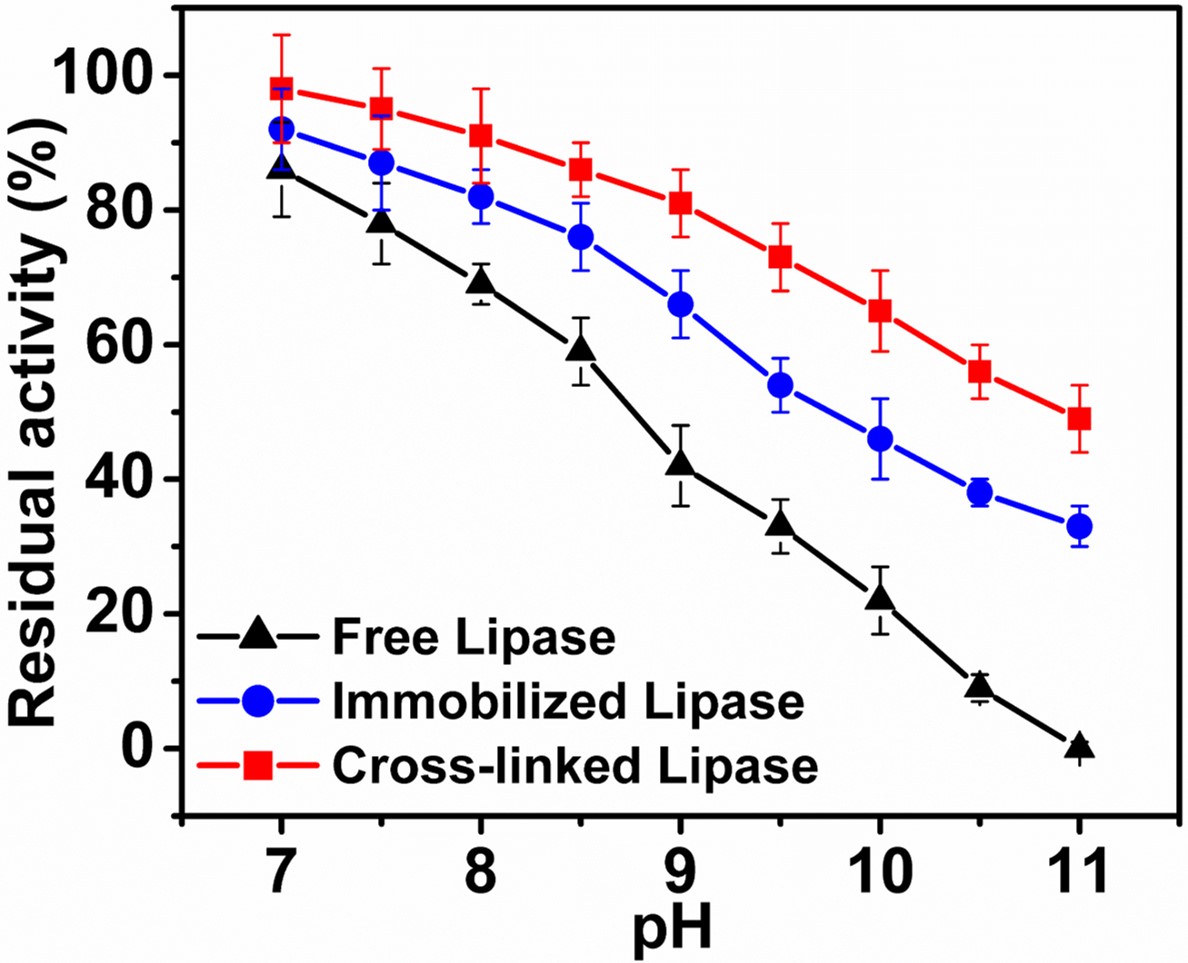


**Supplementary Figure 7.** Stability of free, immobilized, and cross-linked lipase on SnO_2_-nanotubes at different pH(s) from 7 to 11.


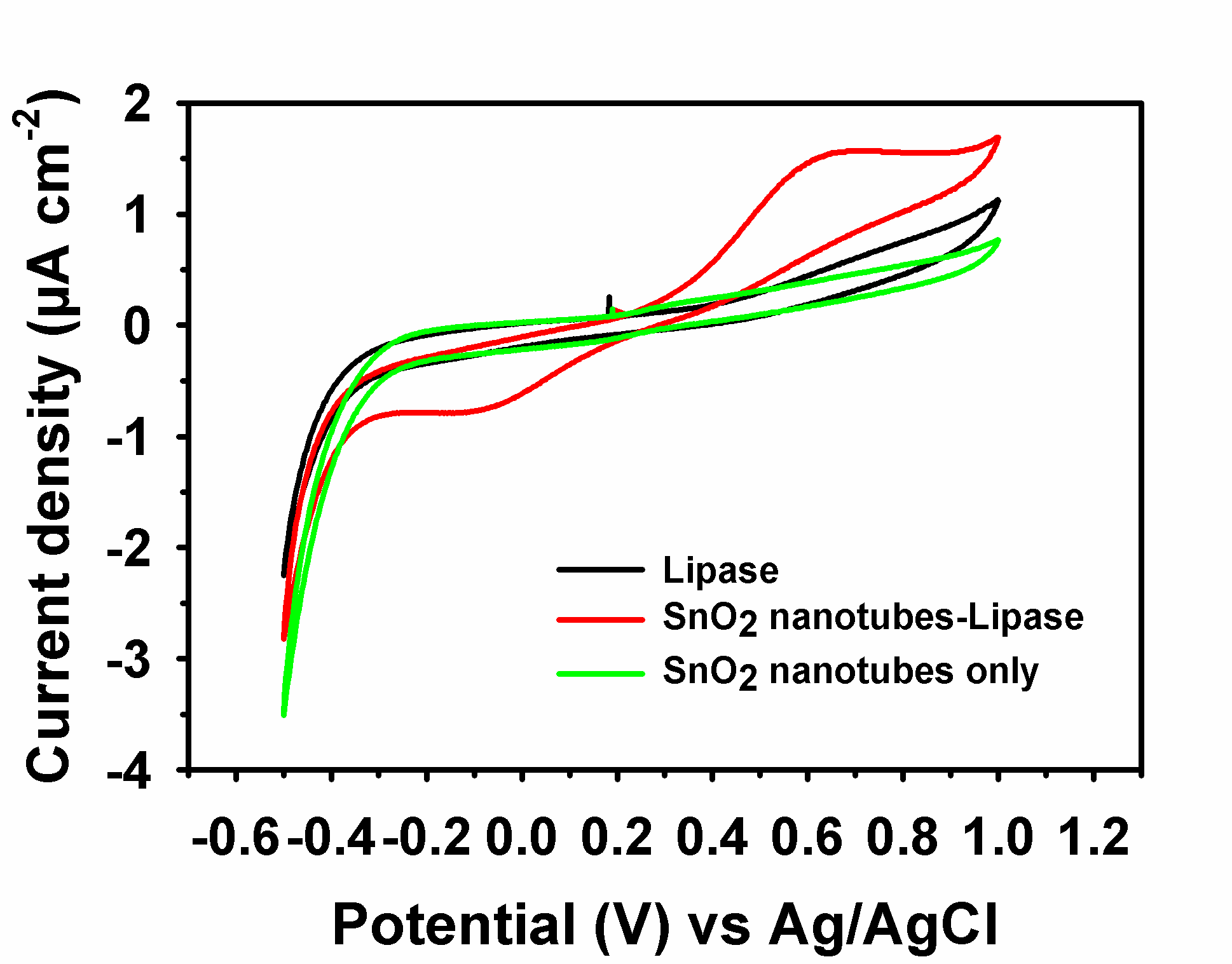


**Supplementary Figure 8.** Electrochemical properties of free lipase, SnO_2_ nanotubes, and lipase immobilized SnO_2_ nanotubes. Cyclic Voltammogram curves of free lipase, SnO_2_ nanotubes, and lipase immobilized SnO_2_ nanotubes in buffer containing palm oil (50 mg/dl).

**Supplementary Table 1.** Immobilization yields (%) and leaching (%) after immobilization and cross-linking of enzymes on SnO_2_-nanotubes.

| Enzyme | IY% | IE% after  immobilization | (%) Leaching  after  immobilization | IE% after  cross-linking | (%) Leaching  after  cross-linking |
| --- | --- | --- | --- | --- | --- |
| Lipase | 93±4 | 89±6 | 36.4±8 | 86±5 | 11.4±4 |
| HRP | 81.5±6 | 78±5 | 32.3±4 | 81±5 | 19.5±2 |
| GOx | 73±5 | 81±6 | 46±5 | 79±5 | 22.5±5 |

**Supplementary Table 2.** Elemental analysis (EA) values given as weight percentage and detection limit as 100 ppm (0.01%).

| NP Type | Nitrogen | Carbon | Oxygen |
| --- | --- | --- | --- |
| Free SnO_2_-NT | 0.00 | 3.60 | 11.03 |
| Immobilized SnO_2_-NT | 1.77 | 11.61 | 18.35 |

**Supplementary Table 3.** Kinetic parameters for the free, immobilized, and cross-linked lipase when enzyme loading was decreased to half of the original loading.

| Lipase | K_m_ (mM) | V_max_ (µmol min^-1^ ml^-1^) |
| --- | --- | --- |
| Free | 0.71 ± 0.05 | 217 ± 20 |
| Immobilized | 0.64 ± 0.08 | 251 ± 30 |
| Cross-linked | 0.72 ± 0.05 | 228 ± 16 |

**Supplementary Table 4.** Effect of different metal ions on relative activity of free and immobilized lipase.

| S. No | Metal ions | Free lipase (RA%) | Immobilized lipase (RA%) |
| --- | --- | --- | --- |
| 1 | Control | 100 | 100 |
| 2 | FeCl_2_ | 98 | 113 |
| 3 | CoCl_2_ | 92 | 106 |
| 4 | MgCl_2_ | 117 | 138 |
| 5 | MnCl_2_ | 131 | 145 |
| 6 | CaCl_2_ | 139 | 162 |
| 7 | FeCl_3_ | 91 | 99 |
| 8 | KCl | 86 | 88 |
| 9 | NaCl | 83 | 92 |
| 10 | NiCl_2_ | 129 | 151 |

**Supplementary Table 5.** Effect of different solvents on relative activity of free and immobilized lipase.

| Organic solvents | Relative activity at 10% (v/v) | | Relative activity at 20% (v/v) | | Relative activity at 30% (v/v) | |
| --- | --- | --- | --- | --- | --- | --- |
| Control | Free | Immobilized | Free | Immobilized | Free | Immobilized |
|  | 100 | 100 | 100 | 100 | 100 | 100 |
| Methyl Formate | 44 | 49 | 32 | 37 | 22 | 29 |
| Methanol | 96 | 100 | 83 | 98 | 77 | 94 |
| n-Hexane | 104 | 112 | 129 | 132 | 138 | 144 |
| Ethanol | 93 | 96 | 90 | 95 | 84 | 93 |
| 10% SDS | 65 | 79 | 45 | 66 | 36 | 52 |
| Propanol | 91 | 97 | 83 | 94 | 76 | 87 |
| Phenol | 32 | 41 | 19 | 32 | 23 | 27 |
| DMSO | 112 | 108 | 115 | 119 | 139 | 168 |
| Acetic acid | 0 | 16 | 0 | 6 | 0 | 0 |
| DMF | 96 | 109 | 117 | 115 | 102 | 117 |
| Acetone | 108 | 112 | 116 | 122 | 131 | 152 |
| Butanol | 77 | 84 | 71 | 78 | 74 | 82 |
